# Effects of SGLT2 ablation or inhibition on corticosterone secretion in high-fat-fed mice: exploring a nexus with cytokine levels

**DOI:** 10.1007/s00125-025-06467-7

**Published:** 2025-06-20

**Authors:** Niki F. Brisnovali, Isabelle Franco, Amira Abdelgawwad, Hio Lam Phoebe Tsou, Thong Huy Cao, John McDonald, Antonio Riva, Guy A. Rutter, Elina Akalestou

**Affiliations:** 1 Section of Cell Biology and Functional Genomics, Division of Diabetes, Endocrinology and Metabolism, Department of Metabolism, Digestion and Reproduction, Imperial College London, London, UK; 2 The Roger Williams Institute of Hepatology, Foundation for Liver Research, London, UK; 3 Faculty of Life Sciences and Medicine, King’s College London, London, UK; 4 Department of Cardiovascular Sciences, College of Life Sciences, University of Leicester, Leicester, UK; 5 National Institute for Health and Care Research Leicester Biomedical Research Centre, University Hospitals of Leicester NHS Trust, Glenfield Hospital, Leicester, UK; 6 Leicester van Geest Multi-OMICS Facility, University of Leicester, Leicester, UK; 7 CHUM Research Centre and Faculty of Medicine, University of Montreal, Montreal, QC Canada; 8 Lee Kong Chian School of Medicine, Nanyang Technological University, Singapore, Singapore

**Keywords:** Corticosterone, Cytokines Glutathione, IL6, SGLT2

## Abstract

**Aims/hypothesis:**

Despite recent therapeutic advances, achieving optimal glycaemic control remains a challenge in managing type 2 diabetes. Sodium–glucose cotransporter 2 (SGLT2) inhibitors have emerged as effective treatments by promoting urinary glucose excretion. However, the full scope of their mechanisms extends beyond glycaemic control. At present, their immunometabolic effects remain elusive.

**Methods:**

To investigate the effects of SGLT2 inhibition or deletion, we compared the metabolic and immune phenotype between high-fat-diet-fed control mice, mice treated chronically with dapagliflozin, and total-body *Slc5a2*-knockout mice.

**Results:**

SGLT2-null mice exhibited better glucose tolerance and insulin sensitivity (blood glucose during IPGTT AUC 0–90 min 1175 ± 57.4 mmol/l × min, mean ± SEM) compared with control (AUC 0–90 min 1857 ± 117.9 mmol/l × min, *p*=0.05) or dapagliflozin-treated mice (AUC 0–90 min 1506 ± 68.72 mmol/l × min, *p*=0.09), independent of glycosuria and body weight. Moreover, SGLT2-null mice demonstrated physiological regulation of corticosterone secretion, with lower morning levels than control mice (*p*<0.01). Systemic cytokine profiling also unveiled significant alterations in inflammatory mediators, particularly IL-6. Furthermore, unbiased proteomic analysis demonstrated downregulation of acute-phase proteins and upregulation of glutathione-related proteins, suggesting a role in the modulation of antioxidant responses. Conversely, IL-6 treatment increased SGLT2 expression in human kidney HK2 cells, suggesting a role for cytokines in the effects of hyperglycaemia.

**Conclusions/interpretation:**

Collectively, our data elucidate a potential interplay between SGLT2 activity, immune modulation and metabolic homeostasis, as well as a potential feedback loop between SGLT2 expression and cytokine concentration.

**Graphical Abstract:**

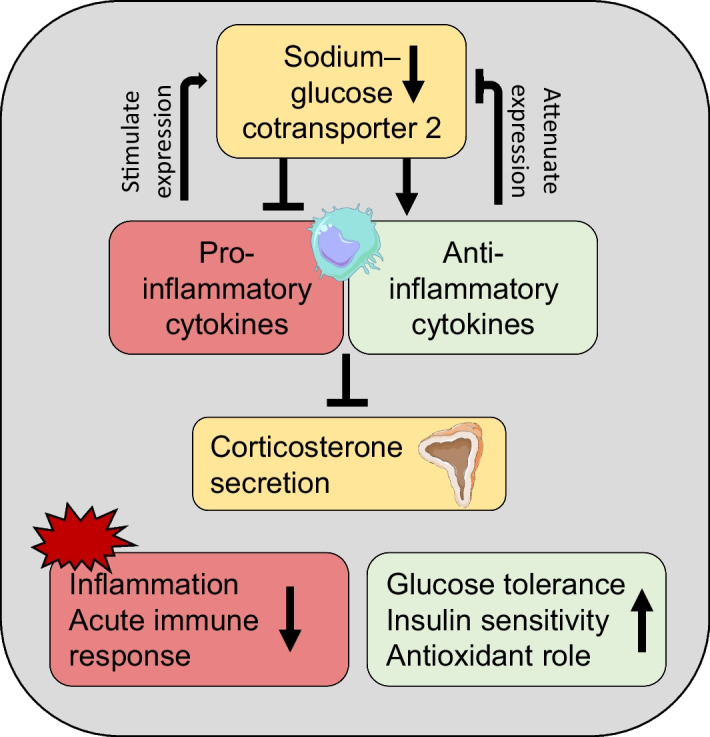

**Supplementary Information:**

The online version contains peer-reviewed but unedited supplementary material available at 10.1007/s00125-025-06467-7.


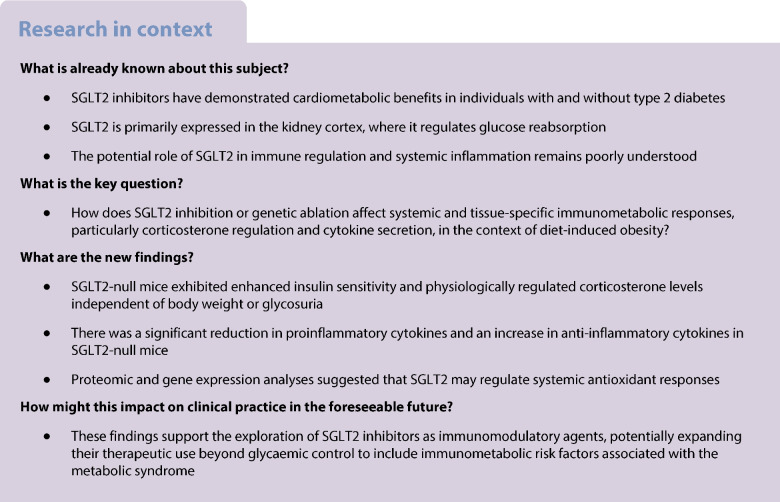


## Introduction

In 2023, the number of people living with diabetes in the UK reached a staggering 5 million, 90% of whom were affected by type 2 diabetes [1]. The National Diabetes Audit (NDA) 2021–2022 quarterly report for England revealed that glucose control deteriorated in individuals with type 2 diabetes, with only 35% meeting the treatment targets for HbA_1c_ [2]. Worldwide, the number of people with type 2 diabetes is predicted to reach 600 million by 2045 [3].

Despite significant pharmacological advances in recent years [4], there is still an unmet need to develop a more effective approach to tackling the pathophysiology of this disease.

Inhibition of sodium–glucose cotransporter 2 (SGLT2) has emerged as an effective means of improving glycaemic control in individuals with type 2 diabetes [5]. SGLT2 is primarily expressed in the kidney cortex and inhibitors, known as gliflozins, block SGLT2 activity in the luminal membrane of the renal early proximal tubule to reduce glucose reabsorption by 50–60% [5]. By lowering the renal glucose threshold to promote glucose excretion in the urine, blood glucose is lowered in an insulin-independent way [5].

SGLT2 inhibitors have been proven to lower HbA_1c_ levels, increase HDL-cholesterol and improve BP, cardiovascular health and kidney disease [5]. Despite their beneficial effects, their exact mechanisms of action are not fully understood and may extend beyond renal glucose reabsorption. In particular, there is growing evidence for a broader, pleiotropic effect of SGLT2 inhibitors on immune regulation [6, 7], including inhibition of T cell activation and proliferation [8] as well as modulation of macrophage M2 polarisation [9]. These findings hint at an intriguing immunometabolic role for SGLT2.

We have recently explored the role of SGLT2 (encoded by *Slc5a2*) in the metabolic responses to high-fat diet (HFD) and metabolic surgery, deploying knockout mice and pharmacological inhibitors [10]. The aim of the present study was to evaluate the role of the immune system in the observed effects of SGLT2 ablation or inhibition.

## Methods

### Animals

All animal procedures were approved by the British Home Office under the UK Animal (Scientific Procedures) Act 1986 with approval from the local ethical committee (Animal Welfare and Ethics Review Board, AWERB), at the Central Biological Services (CBS) Unit at the Hammersmith Campus of Imperial College London. The study was repeated in the CBS at the University of Leicester. Adult male C57BL/6J mice (Envigo, Huntingdon, UK) were maintained under controlled temperature (21–23°C) and a 12 h light–dark cycle (lights on at 07:00 hours).

Mice carrying null *Slc5a2* alleles (*Slc5a2*^−/−^ or ‘SGLT2-null’) lacking exon 1 were generated by us through CRISPR/Cas9-mediated recombination, as previously described [10] in order to compare them with dapagliflozin-treated mice. 11β-Hydroxysteroid dehydrogenase type 1 (11β-HSD1)-null (*Hsd11b1*^−/−^*)* mice, also lacking exon 1, were generated similarly [11] on a C57BL/6J background. In both cases, heterozygous mice were inter-crossed to generate wild-type (WT), heterozygous and homozygous littermates.

Male mice were initially used as a continuation of our previous study [10] to allow direct comparison of results. We also conducted a pilot study using male and female SGLT2-null mice and found no differences in cytokine regulation measurements between the two sexes. From the age of 8 weeks C57BL/6J control and SGLT2-null mice were placed on an HFD with 58% energy from fat and 18.4% from sucrose (D12331, Research Diet, New Brunswick, NJ) to induce obesity and diabetes. Sample size calculation was performed with blood glucose as a primary outcome. Randomisation was performed according to pre-treatment body weight. Group 1 (*n*=8) received 10 mg/kg dapagliflozin dissolved in methylcellulose daily for 14 days, by oral gavage at 08:00 hours. Masking was not carried out during this study. No animals were excluded during the study. Biopsies (kidneys, adipose tissue, plasma) were harvested from all mice in the fed state, and were snap-frozen at −80°C.

### GTTs

Mice were fasted for 8 h and given free access to water. Blood was sampled in EDTA-coated tubes from the tail vein at 0, 5, 15, 30, 60 and 90 min after i.p. glucose administration (1 g/kg at 15:00 hours). For the measurement of total glucagon-like peptide 1 (GLP-1), glucose (3 g/kg body weight) was administered via oral gavage (08:00 hours) following overnight (total 16 h) fasting and aprotinin was added in the collection tubes. Blood glucose was measured with an automatic glucometer (Accuchek; Roche, Burgess Hill, UK).

### ITTs

Mice were fasted for 6–8 h (starting at 08:00 hours) and given free access to water. At 15:00 hours, human insulin (Actrapid, Novo Nordisk) (1.5 U/kg body weight) was administered via i.p. injection. Blood was sampled as described for GTTs.

### Urine glucose and creatinine measurement

Urine was collected from mice during GTTs by placing them in a cage without bedding. Glucose was measured by Glucose Assay (Abcam, USA) and creatinine was measured by Creatinine Assay (Crystal Chem, IL, USA). The urinary glucose/creatinine ratio (UGCR) was calculated in μmol/μmol (urine glucose [μmol/l]/urine creatinine [μmol/l]).

### Cell culture and treatments

Cells were cultured with the aim of measuring *SLC5A2* expression in response to cytokine treatment. Human kidney-2 (HK2) cells were purchased from ATCC (Manassas, VA, USA) and were cultured with Keratinocyte Serum Free Medium (K-SFM) supplemented with 0.05 mg/ml bovine pituitary extract (BPE) and 5 ng/ml EGF (Invitrogen, USA), without added glucose. Mycoplasma-free certificate was provided by the company. Cells were seeded at a density of 1 × 10^5^ per well and treated for 72 h with glucose (30 mmol/l), IL-6 (1 nmol/l), leptin (5 nmol/l), fibroblast growth factor-21(FGF-21) (10 nmol/l), IL-1β (1 nmol/l) or TGF-β1 (400 pmol/l) (Abcam, USA). Four to ten different replicates were used for each condition.

NCI-H295R cells (a human adrenocarcinoma cell line) were obtained from ATCC (Manassas, VA, USA) and cultured in DMEM:F12 medium, supplemented with 0.00625 mg/ml insulin, 0.00625 mg/ml transferrin, 6.25 ng/ml selenium, 1.25 mg/ml BSA, 0.00535 mg/ml linoleic acid and 2.5% Nu-Serum (Corning, USA). Cells were seeded at a density of 1 × 10^5^ per well and treated for 24 h with IL-6 (1 nmol/l), leptin (5 nmol/l), FGF-21 (10 nmol/l), IL-1β (1 nmol/l), TGF-β1 (400 pmol/l) or IL-10 (1 nmol/l) (Abcam, USA). Four to ten different replicates were used for each condition.

### RNA isolation, cDNA synthesis and quantitative PCR

RNA was extracted from cell and tissue samples by resuspending them in TRIzol reagent (Ambion, Life Technologies, USA) and chloroform (Sigma-Aldrich, USA). Total RNA isolation was conducted using the TRIzol Plus RNA Purification Kit (Applied Biosystems, ThermoFisher, USA) according to the manufacturer’s protocol. The High-Capacity cDNA Reverse Transcription kit (Applied Biosystems, ThermoFisher, USA) was used for cDNA synthesis. Master mix for quantitative PCR (qPCR) was made using SYBR Green (Applied Biosystems, ThermoFisher, USA), RNase samples were analysed using the 7500 Fast Real-Time PCR system (Applied Biosystems, serial no. 275010654). All data were normalised against β-actin as the housekeeping gene and all ∆C_t_ values were calculated using the average from dual replicates. The ${2}^{{-\Delta \Delta \text{C}}_{\text{t}}}$ analytical method was used. The primers used are shown in electronic supplementary material (ESM) Table 1.

### ELISAs

Blood plasma and urine protein concentrations of corticosterone (Crystal Chem), GLP-1 (Crystal Chem, IL, USA), aldosterone (Crystal Chem, IL, USA) and TGF-β1 (Proteintech, USA) were measured using their respective assay kits and processed according to manufacturer’s protocol.

### Multiplex cytokine quantification

Blood was collected at the end of treatment using a cardiac puncture. The samples were centrifuged at 10,000 *g* for 8 min at 4°C to isolate the plasma. The blood plasma was stored at −20°C. Blood plasma cytokine concentrations were measured using a Luminex Mouse Discovery Assay (8-Plex) (LXSAMSM-07, Bio-techne, USA) according to manufacturer’s instructions. Standard curves were interpolated by five-parameter logistic curves. All quantified values between sensitivity limits and lowest standard points were included in the analysis. All values below quantification limits were also included in the analysis as sensitivity or ‘half-minimum’ values.

### Proteomics analysis

Proteomics analysis was conducted by Seer (USA) through LC-MS.

#### Sample preparation for direct digest protocol

For direct digestion of neat plasma samples, proteins were denatured, reduced, alkylated and subjected to proteolytic digestion (trypsin and Lys-C) for 3 h at 37°C. Peptides were purified by solid-phase extraction and yields were determined (Applied Biosystems, ThermoFisher, USA).

#### Sample preparation with the Proteograph XT assay protocol

Samples were processed with the Proteograph XT assay [12]. In brief, plasma proteins were quantitatively captured in nanoparticle (NP)-associated protein coronas. Proteins were subsequently denatured, reduced, alkylated, and subjected to proteolytic digestion (trypsin and Lys-C). Peptides were purified and yields were determined. Peptides were dried down overnight with a vacuum concentrator and reconstituted with a reconstitution buffer to a concentration of 50 ng/µl.

#### Data-independent acquisition LC-MS/MS

For data-independent acquisition (DIA), 8 µl of reconstituted peptide mixture from each NP preparation was analysed resulting in a constant 400 ng MS injection between NP A and NP B samples. Each sample was analysed with a Vanquish NEO nanoLC system coupled with a Orbitrap Astral (Applied Biosystems, ThermoFisher, USA) mass spectrometer using a trap-and-elute configuration. First, the peptides were loaded onto an Acclaim PepMap 100 C18 (0.3 mm ID × 5 mm) trap column and then separated on a 50 cm µPACTM analytical column (PharmaFluidics, ThermoFisher, USA) at a flow rate of 1 µl/min using a gradient of 5–25% solvent B (0.1% formic acid [FA], 100% acetonitrile [ACN]) mixed into solvent A (0.1% FA, 100% water) over 22 min, resulting in a 30 min total run time. The mass spectrometer was operated in DIA mode with MS1 scanning and MS2 precursor isolation windows between 380 m/z and 980 m/z. MS1 scans were performed in the Orbitrap detector at 240,000 R every 0.6 s with a 5 ms ion injection time or 500% Automatic Gain Control (AGC) (500,000 ion) target. Two hundred fixed-window MS2 DIA scans were collected at the Astral detector per cycle with 3Th precursor isolation windows, 25% normalised collision energy and 5 ms ion injection times with a 500% (50,000 ion) active gain control maximum. MS2 scans were collected from 150 to 2000 m/z.

### Statistical analysis

Each single mouse was used as a unit of analysis (*n*=5 mice per group). Each cell culture experiment was repeated at least three times. Principal component analysis (PCA) was performed to determine if there was a good separation between the three mouse groups (HFD vs dapagliflozin treatment vs SGLT2-null) using SIMCA version 14 (MSK Umetrics). The fold change and adjusted *p* values in comparisons between the three mouse groups were calculated using Limma R package (https://posit.co/download/rstudio, Posit, RStudio/2024.12.1+563) with adjustment for Benjamini–Hochberg (BH) false discovery rate (FDR) of 0.05 to control multiple testing errors and then volcano plots were generated. Data were analysed using GraphPad Prism software (https://www.graphpad.com/, GraphPad, GraphPad 10). Comparisons between two groups were carried out using Mann–Whitney test. Group comparisons were analysed using a two-way ANOVA, given lack of missing measurements, with Geisser–Greenhouse correction and Sidak’s multiple comparison test. The model included main effects and interaction, to be able to evaluate group differences at individual timepoints. All statistical tests were two-tailed, and significance was set at α=0.05. Data are presented as median (IQR).

### Data and resource availability

All data generated during this study are included in the published article (and its online supplementary files).

## Results

### SGLT2-null mice display improved glucose tolerance compared with dapagliflozin-treated mice independent of glycosuria levels

**Fig. 1:**
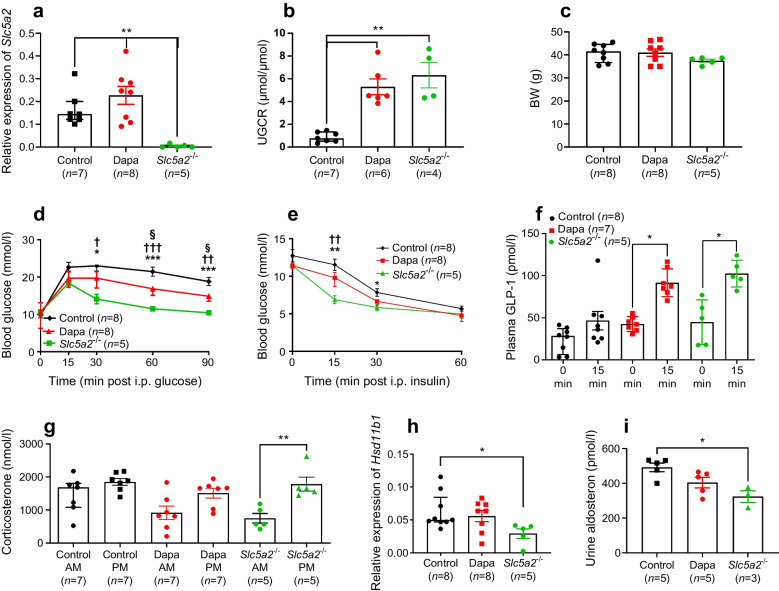
(**a**) Gene expression of *Slc5a2* relative to *Actb* in kidney of WT HFD-fed mice following 2 weeks of treatment with methylcellulose (Control, black) or dapagliflozin (10 mg/kg) (Dapa, red) by oral gavage and HFD-fed SGLT2-null obese mice (green, *Slc5a2*^−/−^). (**b**) UGCR post-IPGTT (1 g/kg, 8 h fasting). (**c**) Body weight. (**d**) Blood glucose levels following IPGTT (1 g/kg, 8 h fasting). (**e**) Blood glucose levels following ITT (1.5 U/kg, 6–8 h fasting). (**f**) GLP-1 levels following oral GTT (3 g/kg, 16 h fasting). (**g**) Plasma corticosterone concentration in the morning (08:00–09:00 hours; AM) and evening (17:00–18:00 hours; PM). (**h**) Gene expression (mRNA levels) of *Hsd11b1*, relative to *Actb* in the subcutaneous adipose tissue. Liver from one mouse did not produce adequate RNA quality over three attempts and was excluded. (**i**) Urine aldosterone measured in the fed state in the morning. (**a**, **b**, **f**–**i**) *p<0.05, **p<0.01 as shown. (**d**, **e**) ^†^*p*<0.05, ^††^*p*<0.01, ^†††^*p*<0.001, WT vs *Slc5a2*^−/−^; *p<0.05, **p<0.01, ***p<0.001, WT+Dapa vs *Slc5a2*^−/−^; ^§^*p*<0.05, WT+Dapa vs WT. BW, body weight

Whole-body *Slc5a2-*knockout mice and WT littermate controls were exposed to 12 weeks of HFD. Subsequently, WT mice were treated with either dapagliflozin (10 mg/kg) or control vehicle (methylcellulose) via oral gavage daily for 14 days. To confirm deletion, levels of *Slc5a2* were measured in kidney cortex biopsies in all mice (Fig. 1a). Of note, *Slc5a2* levels were significantly upregulated in dapagliflozin-treated mice compared with untreated WT mice. Glucose/creatinine ratio measurement in the urine of mice following a 3 g/kg glucose load showed no significant difference between the two intervention groups (Fig. 1b).

No significant weight loss was observed in either group (Fig. 1c). SGLT2-null mice had a lower body weight (37.5 ± 1.6 g) than the dapagliflozin- and vehicle-treated mice (41 ± 4.5 g and 40.9 ± 3.9 g, *p*=0.10 and *p*=0.2 respectively). During IPGTTs (1 g/kg) SGLT2-null mice displayed significantly improved glucose clearance (blood glucose total AUC 0–90 min 1175 ± 57.4 mmol/ l × min, mean ± SD) when compared with control WT (blood glucose total AUC 0–90 min 1857 ± 117.9 mmol/ l × min) and dapagliflozin-treated (blood glucose total AUC 0–90 min 1506 ± 68.72 mmol/ l × min) WT mice (Fig. 1d). SGLT2-null mice also showed improved insulin tolerance (Fig. 1e).

To investigate whether these differences may reflect higher levels of the incretin GLP-1, plasma levels of GLP-1 were measured after 16 h of fasting and following glucose oral gavage (3 g/kg). Compared with untreated mice, glucose-induced GLP-1 increases were restored in SGLT2-null mice (*p*<0.05, 0 vs 15 min, *n*=5) and dapagliflozin-treated WT mice (*p*<0.05, *n*=5, 0 vs 15 min) mice vs HFD-fed control mice (Fig. 1f).

### Corticosterone secretion and *Hsd11b1* expression is inhibited in SGLT2-null mice

To further explore the mechanisms behind the significantly improved glucose clearance and insulin tolerance in SGLT2-null mice, and more minor effects on these variables in dapagliflozin-treated WT mice, we measured the levels of corticosterone in the morning and evening in plasma (Fig. 1g). Obese WT mice demonstrated high levels of corticosterone in the morning followed by a marginal postmeridian increase, consistent with studies reporting increased levels of corticosterone (cortisol in humans) in obesity [13]. In contrast, SGLT2-null mice on the same diet demonstrated a more physiological secretion pattern, reflecting tendencies towards both lowered morning and increased evening corticosterone levels (Fig. 1g). Correspondingly, expression of *Hsd11b1* (encoding the corticosterone-activating enzyme 11βHSD1) in subcutaneous adipose tissue (Fig. 1h) was significantly reduced in SGLT2-null mice (*p*=0.015) but was unaffected in dapagliflozin-treated mice. Of note, urine levels of aldosterone were significantly inhibited in SGLT2-null mice (Fig. 1i).

### SGLT2 expression and glycosuria are unaffected in 11βHSD1-null mice

**Fig. 2:**
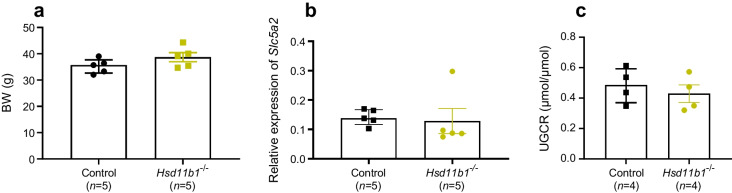
(**a**) Body weight of HFD-induced obese WT mice (Control) and 11βHSD1-null obese mice (*Hsd11b1*^−/−^). (**b**) Gene expression (mRNA levels) of *Slc5a2* relative to *Actb* in kidney. (**c**) UGCR post-IPGTT (3 g/kg). BW, body weight

To explore the possibility of a bidirectional relationship between SGLT2 and 11βHSD1 expression, we used total-body *Hsd11b1*-knockout mice [11] and assessed *Slc5a2* expression and function after 12 weeks on HFD. No significant differences in body weight were observed between the knockout mice and control mice (Fig. 2a). No difference in *Slc5a2* expression in the kidney cortex (Fig. 2b) and no difference in the glucose/creatinine ratio (Fig. 2c) was observed.

### SGLT2-null mice demonstrate altered circulating cytokine levels in plasma and tissue

**Fig. 3:**
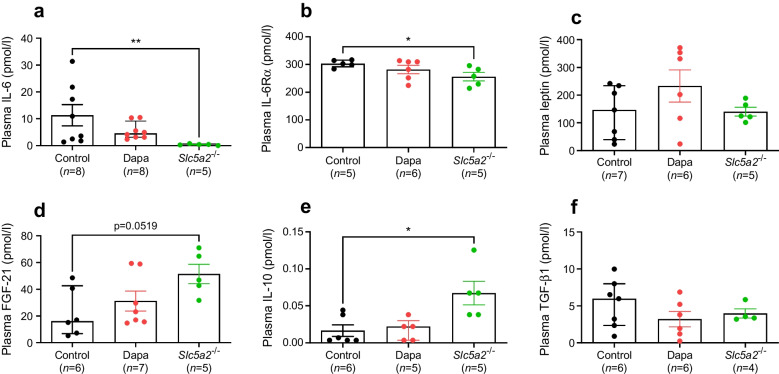
Plasma concentration of IL-6 (**a**), IL-6Rα, (**b**), leptin (**c**), FGF-21 (**d**), IL-10 (**e**) and TGF-β1 (**f**) in HFD-induced obese WT mice following 2 weeks of treatment with methylcellulose (Control) or dapagliflozin (10 mg/kg, Dapa) and in SGLT2-null obese mice (*Slc5a2*^−/−^). *p<0.05, **p<0.01, as shown

**Fig. 4:**
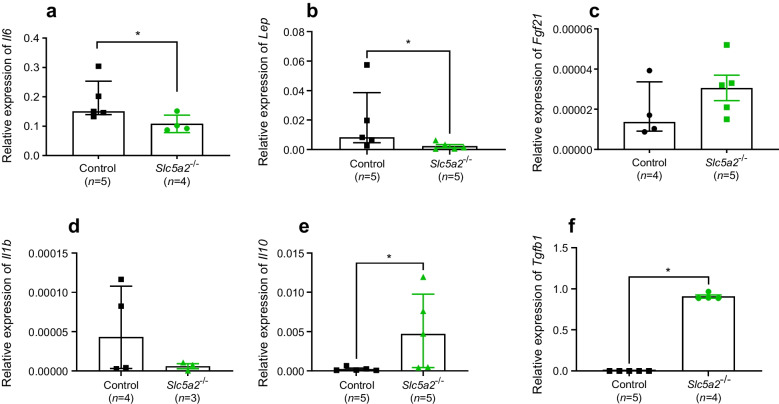
Gene expression (mRNA levels) of *Il6* (**a**), *Lep* (**b**), *Fgf21* (**c**), *Il1b* (**d**), *Il10* (**e**) and *Tgfb1* (**f**) relative to *Actb* in the subcutaneous adipose tissue of HFD-induced obese WT and SGLT2-null mice. *p<0.05, as shown

Following our finding that corticosterone secretion is normalised in SGLT2-null mice, we sought next to investigate whether the effects of *Sglt2* deletion or SGLT2 inhibition on glucose homeostasis and corticosterone levels may involve altered levels of cytokines. Levels of the proinflammatory cytokine IL-6 and the α subunit of its receptor complex (IL-6Rα) were significantly lowered (*p*=0.0079 and *p*=0.0159) in SGLT2-null mice (Fig. 3a, b). In contrast, levels of the anti-inflammatory cytokines FGF-21 and IL-10 were increased compared with levels in WT mice (*p*=0.01 and *p*=0.0519, respectively) (Fig. 3d, e). TGF-β1 levels remained unchanged (Fig. 3f). Of note, IFN-γ, TNF-α and IL-1β were always below detection range. To verify these findings, we measured the gene expression of the most prominent cytokines in subcutaneous adipose tissue biopsies of the same SGLT2-null and WT mice. Both *Il6* and *Lep* genes were downregulated in SGLT2-null mice, while *Il10* and *Tgfb1* were upregulated (Fig. 4) vs WT mice.

### Circulating levels of proteins involved in inflammation were downregulated in both dapagliflozin-treated and SGLT2-null mice

**Fig. 5:**
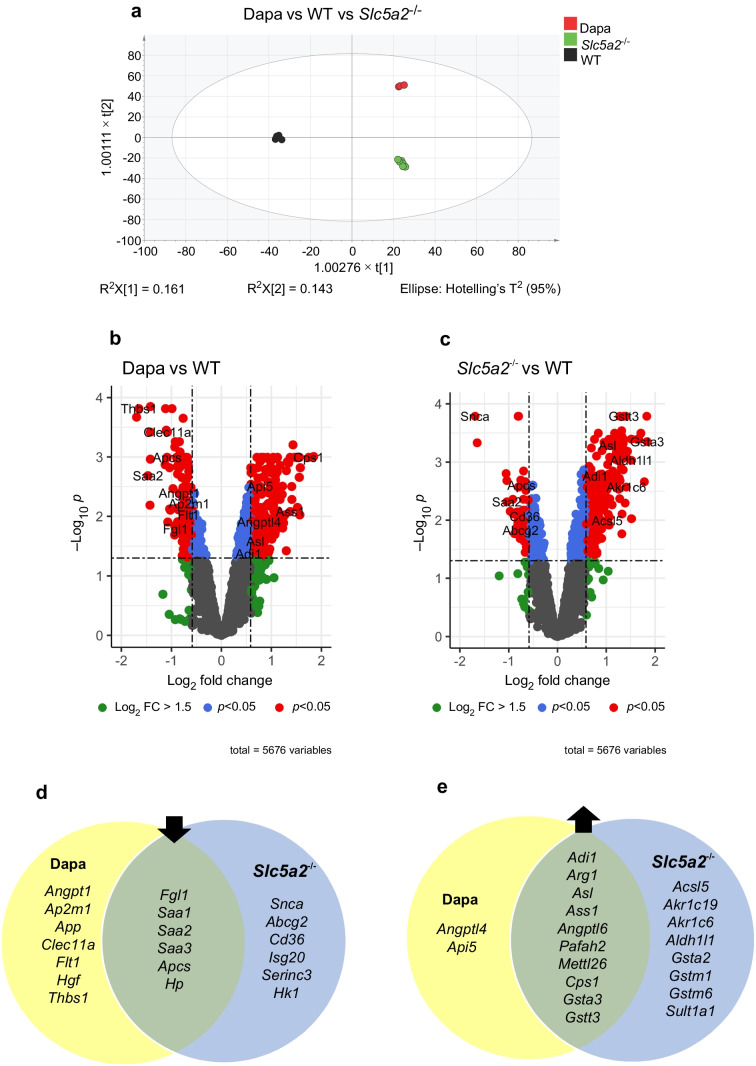
(**a**) PCA plot of the three groups: HFD-induced obese WT mice following 4 weeks of methylcellulose (WT, black,* n*=6) or dapagliflozin treatment (10 mg/kg) (Dapa, red, *n*=3); and SGLT2-null obese mice (*Slc5a2*^−/−^, green, *n*=6). R^2^X represents the proportion of variance in the data explained by the principal components, Hotelling’s T^2^ charts, are used for multivariate outlier detection and process monitoring in data with high dimensionality and correlation. (**b**, **c**) Volcano plots showing genes associated with differentially abundant plasma proteins in dapagliflozin-treated mice vs WT mice (**b**) and SGLT2-null mice vs WT mice (**c**). (**d**) Venn diagram displaying genes associated with immune response-related proteins downregulated in dapagliflozin-treated (yellow) or SGLT2-null (blue) mice, or both (green). (**e**) Genes associated with immune response-related proteins upregulated in dapagliflozin-treated (yellow) or SGLT2 null (blue) mice, or both (green). FC, fold change

Plasma from the three mouse groups was further analysed using MS. PCA showed clear difference between the groups (Fig. 5a). We quantified 5660 plasma proteins, 127 of which were downregulated and 252 were upregulated in dapagliflozin-treated mice, when compared with WT control mice (Fig. 5b). In the SGLT2-null mice, 57 proteins were downregulated and 233 were upregulated, when compared with WT control (Fig. 5c). Of these proteins, 18 were downregulated and 92 were upregulated in both dapagliflozin-treated and SGLT2-null mice. We narrowed the targets by focusing on the proteins with the highest fold change (>1.5-fold change) and lowest *p* value threshold (*p*<0.05). Metascape version v3.5.20240101 was employed for gene ontology (GO) and pathway enrichment as well as protein interaction network analyses (https://metascape.org). Both dapagliflozin-treated and SGLT2-null mice demonstrated significant downregulation of six proteins involved in acute liver response to possible immune challenge and immune system processes: fibrinogen-like protein 1; amyloid P component; haptoglobin; serum amyloid A1; serum amyloid A2; and serum amyloid A3 (Fig. 5d). Four proteins involved in vessel morphogenesis were downregulated in dapagliflozin-treated mice: thrombospondin 1; Fms-related tyrosine kinase 1; hepatocyte growth factor; and angiopoietin 1 (Fig. 5d). Six metabolism and immune response proteins were downregulated in SGLT2-null mice alone, including synuclein α (SNCA) [14], a hallmark in neurodegenerative disorders, the glucose phosphorylating enzyme, hexokinase 1 (HK1), the transmembrane glycoprotein CD36, and IFN stimulated exonuclease gene 20 (ISG20) [15], involved in the innate immune response.

### SGLT2-null mice exhibit upregulated expression of proteins involved in glutathione production

Of the 92 proteins found to be upregulated in both dapagliflozin-treated and SGLT2-null mice, four (argininosuccinate synthase 1, arginase 1, argininosuccinate lyase, carbamoyl-phosphate synthase 1) were enzymes involved in the urea cycle (Fig. 5e), as well as in cellular responses to glucagon. Importantly, three proteins involved in ‘glutathione metabolic process’ were upregulated in both groups, compared with WT mice (glutathione *S*-transferase α3, glutathione *S*-transferase θ3, glutathione *S*-transferase μ3) (Fig. 5e), while SGLT2-null mice displayed significantly upregulated expression of eight additional glutathione-regulating proteins.

### Differentially expressed protein classification and functional enrichment analysis

**Fig. 6:**
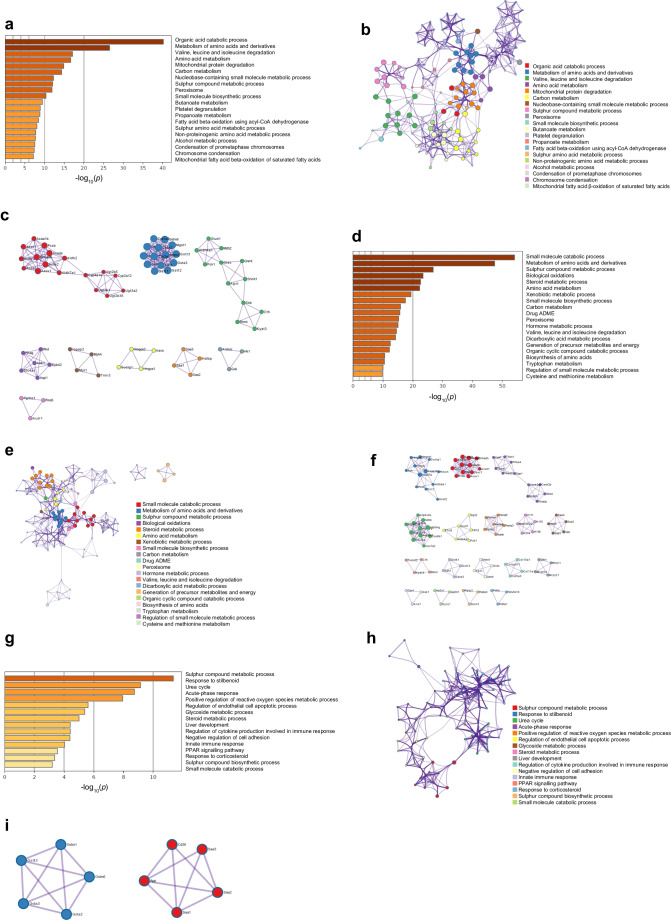
(**a**) Bar plot of enriched pathways of DEPs in SGLT2-null mice vs WT mice. (**b**) Clustered networks of the enriched terms for the DEPs. (**c**) Protein–protein interaction networks for the DEPs. (**d**) Bar plot of enriched pathways of DEPs in dapagliflozin-treated mice vs WT mice. (**e**) Clustered networks of the enriched terms for the DEPs. (**f**) Protein–protein interaction networks for the DEPs. (**g**) Bar plot of enriched pathways of immune response-related DEPs in both SGLT2-null and dapagliflozin-treated mice vs WT mice. (**h**) Clustered networks of the enriched terms for the immune response-related DEPs in both SGLT2-null and dapagliflozin-treated mice. (**i**) Protein–protein interaction networks for the DEPs. ADME, absorption, distribution, metabolism and excretion; PPAR, peroxisome proliferator-activated receptor

The proteins differentially expressed in SGLT2-null mice were significantly enriched in 20 biological pathways and high-level GO terms (Fig. 6a). The enrichment network and protein–protein interaction analysis showed that their functions were clustered in the biological process of small molecule catabolic process and the metabolism of amino acids and their derivatives (Fig. 6b, c). The enrichment network of differentially expressed proteins (DEPs) in dapagliflozin-treated mice showed similar function results, with the notable addition of mitochondrial protein degradation (Fig. 6d–f). Function analysis of DEPs in both SGLT2-null and dapagliflozin-treated mice (Fig. 5d, e) primarily showed an increase in proteins involved in sulphur metabolism and response to stilbenoid (Fig. 6g–i).

### Cytokines directly alter SGLT2/SLC5A2 expression in human kidney cells

**Fig. 7:**
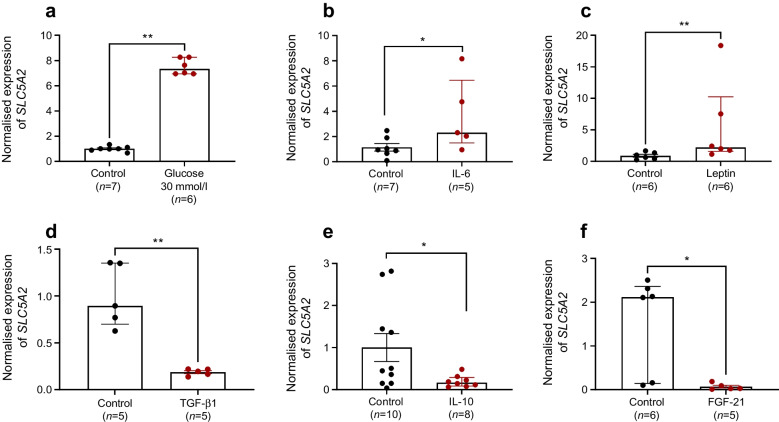
(**a**) Normalised gene expression (mRNA levels) of *SLC5A2* relative to *ACTB* in human kidney HK2 cell lysates following 96 h of treatment with glucose (30 mmol/l) (**a**), IL-6 (1 nmol/l) (**b**), leptin (5 nmol/l) (**c**), TGF-β1 (400 pmol/l) (**d**), IL-10 (1 nmol/l) (**e**) or FGF-21 (10 nmol/l) (**f**), compared with vehicle treatment (Control). Normalisation against vehicle-treated cells. *p<0.05, **p<0.01, as shown

To determine whether increased SGLT2 expression may involve inflammatory mediators, we treated human kidney cells (HK2) with a selection of the cytokines flagged during the in vivo experiments above (Figs 3, 4). The expression in HK2 cells of the cognate receptor for each cytokine was confirmed by qPCR. The cell line was first treated with a high concentration of glucose (30 mmol/l) and revealed a dose-responsive expression of *SLC5A2* (Fig. 7a). Treatment with proinflammatory cytokines IL-6 and leptin (corresponding to the proteins with reduced gene expression in SGLT2-null mice [Fig. 4a, b]) also strongly induced *SLC5A2* expression (Fig. 7b, c), compared with untreated control. Conversely, cells treated with the anti-inflammatory cytokines TGF-β1, IL-10 and FGF-21 (corresponding to proteins showing increased gene expression in SGLT2-null mice [Fig. 4c, e, f]) displayed significantly lower *SLC5A2* expression (Fig. 7d, e, f).

### Proinflammatory cytokines increase the expression of key cortisol-regulating enzymes

**Fig. 8:**
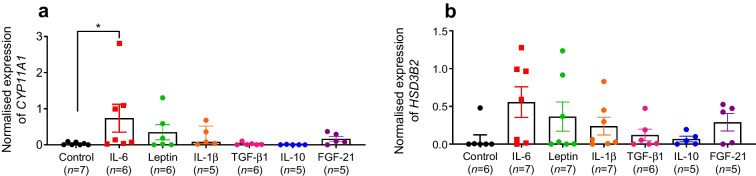
Normalised gene expression (mRNA levels) of *CYP11A1* (**a**) and *HSD3B2* (**b**) relative to *ACTB* in human adrenal gland H295R cells following 24 h of treatment with IL-6 (1 nmol/l), leptin (5 nmol/l), IL-1β (1 nmol/l), TGF-β1 (400 pmol/l), IL-10 (1 nmol/l) or FGF-21 (10 nmol/l), compared with vehicle treatment (Control). Normalisation against vehicle-treated cells. *p<0.05

To explore the possibility that altered cytokine levels (Figs 3, 4) may contribute to the apparent normalisation of corticosterone concentration after *SGLT2* deletion (Fig. 1g, h), we treated the human adrenal cortex cell line H295R with the previously identified cytokines (Figs 3, 4, 5). After 24 h of treatment, the proinflammatory cytokine IL-6 (and, to a lesser extent, leptin) increased the expression levels of the cortisol synthesis enzymes encoded by *CYP11A1* and *HSD3B2.* The anti-inflammatory cytokines TGF-β1, IL-10 and FGF-21 had no effect (Fig. 8a, b).

## Discussion

We show here that SGLT2-null mice on an HFD have improved glucose tolerance and insulin sensitivity, compared with dapagliflozin-treated mice. This effect appears to be independent of glycosuria and may be due to a reduction in proinflammatory cytokine secretion, resulting in downregulation of immune response-regulating corticosterone production, as indicated by our in vitro study.

Our study demonstrated modest differences in weight and blood glucose levels between control and dapagliflozin-treated mice, though significant differences were present in SGLT2-null vs WT mice. The glycosuria levels of dapagliflozin-treated and SGLT2-null mice were similar, Human studies, such as the CREDENCE study [16] and its sub-analyses [17, 18], also reported very modest differences between canagliflozin-treated and control groups, with regards to blood glucose levels, weight and BP, although the authors hypothesised that the observed effects were a result of reduction in intraglomerular pressure and also other unexplored mechanisms. To explore further mechanisms in our study, we measured corticosterone levels. Corticosterone (cortisol in humans) is a steroid hormone secreted primarily by the adrenal glands. Excess cortisol and high levels of its activating enzyme 11βHSD1 are linked to profound metabolic disturbances resulting in insulin resistance [13], as well as increased production of inflammatory cytokines [19]. Obesity has often been associated with cortisol dysregulation and excess secretion [13]. We show that complete ablation of SGLT2 restores a normal ‘physiological’ fluctuation [11] in corticosterone in HFD-fed mice, an effect which also was observed, albeit to a lesser extent, after dapagliflozin treatment. The SGLT2 inhibitor empagliflozin has been reported to improve chronic hypercortisolism-induced abnormal myocardial structure and cardiac function in mice [20]. Similarly, tofogliflozin is reported to reduce cortisol levels through reduction of ACTH, in individuals with type 2 diabetes [21]. This does not appear to be the driving mechanism in our study, as proteomics analysis measurements of ACTH secretion plasma markers (pro-opiomelanocortin [POMC], proprotein convertase subtilisin/kexin type 1 [PCSK1], proprotein convertase subtilisin/kexin type 2 [PCSK2], serpin family A member 6 [SERPINA6]) in SGLT2-null and dapagliflozin-treated mice revealed no significant differences when compared with control mice. The observation that 11βHSD1-null mice have normal levels of SGLT2 gene expression and glycosuria (Fig. 2), indicates that the reduced corticosterone in SGLT2-null mice is likely an outcome, rather than a part of a feedback loop (Fig. 1g, h).

We also observed significantly lower levels of in the proinflammatory cytokine IL-6 and IL6Rα in SGLT2-null mice, compared with the two WT groups. Although dapagliflozin-treated mice did not achieve significantly lower IL-6 concentrations, as previously described [22, 23], they did display lower levels than vehicle-treated control mice. Leptin levels remained comparable with those in control mice in both dapagliflozin-treated and SGLT2-null mice. Previous studies have shown that pharmacological inhibition of SGLT2 reduces leptin levels [24]. However, this effect was linked to SGLT2-inhibitor therapy-induced weight loss, an effect that was absent in the present study after dapagliflozin treatment. This may explain the overall lack of change in cytokine levels in dapagliflozin-treated mice. We note that it has been previously shown that, maintained on a high diet, SGLT2-null mice did not display significant weight loss when compared with WT control mice [25]. The anti-inflammatory cytokines FGF-21 and IL-10, known for their roles in improving insulin sensitivity [26] and inhibiting inflammation [27], were present at higher circulating levels in SGLT2-null mice vs control WT mice, while TGF-β1 concentration was unchanged.

We further evaluated these findings in SGLT2-null mice by measuring the expression of cytokine genes in subcutaneous adipose tissue. Our findings on plasma levels of IL-6, FGF-21 and IL-10 were mirrored by altered gene expression in adipose tissue. Additionally, leptin gene expression was significantly reduced in SGLT2-null mice, while *Tgfb1* was increased. This could indicate a more tissue-specific role, or it may be related to the lack of association between gene expression and plasma cytokine levels. Leptin is primarily produced in adipose tissue and high levels are closely associated with insulin resistance and inflammation [28]. The role of TGF-β1 is less clear. It can be produced, secreted and stored in the extracellular matrix without functional consequences until tissue remodelling processes are induced [29]. In adipose tissue, TGF-β1 has been shown to be upregulated in obese mice [30]. However, its role in the regulation of inflammatory responses depends on the differentiation stage of adipocytes [31, 32], while the TGF-β1 receptor has been shown to regulate progenitors that promote browning of white fat [33]. As SGLT2-null mice did not demonstrate significantly different body weight compared with WT controls, it is likely that the increase in TGF-β1 is linked to the latter effect.

Upon analysis of plasma proteomes in the three mouse groups, we found that both dapagliflozin-treated and SGLT2-null mice had reduced levels of acute-phase proteins, which are produced by the liver in response to proinflammatory stimuli. Fibrinogen-like protein 1 (FGL1) is considered an immune checkpoint due to its role in modulating T cell function [34]. Haptoglobin is an acute-phase protein that is found to be elevated in several inflammatory diseases [35]. Similarly, serum amyloid A proteins have a critical role in the regulation and possibly propagation of an initial acute-phase response [36]. As expected, both interventions resulted in the upregulation of enzymes involved in the urea cycle, linked to the acute contraction in plasma volume caused by SGLT2 inhibition [37]. Additionally, upregulation of enzymes linked to glucagon cell response was detected, following our previous finding of increased glucagon secretion in mouse plasma, post-dapagliflozin treatment only [10]. Of note, stilbenoid responding proteins, sulphur compound metabolism-regulating proteins and glutathione-regulating proteins were also found to be differentially expressed in both groups, compared with control, especially in SGLT2-null mice. Glutathione regulation has an indirect antioxidant role and has recently been reported for SGLT2 inhibitors as a potential mechanism of their ability to reduce high-glucose-induced oxidative stress [38, 39]. Stilbenoids are antimicrobial compounds with anti-inflammatory action that exert various other biological activities such as cardioprotection and glucose-lowering properties [40], while sulphur metabolism regulates the inflammatory process of macrophages and creates a negative feedback loop to limit excessive inflammation [41].

Providing evidence of a role for altered cytokine levels in the observed normalisation of corticosterone fluctuations in SGLT2-null mice, treatment of adrenal gland cells with IL-6 upregulated *CYP11A1* and *HSD3B2*, in line with previous findings [42]. Nevertheless, the direct demonstration of a role for inflammatory cytokines in mediating the effects of SGLT2 deletion (or inhibition) in the living animal will require inactivation of the corresponding cytokine or receptor genes, or immunostasis, which lie beyond the scope of the present study.

We also demonstrate that proinflammatory cytokines IL-6 and leptin caused an increase in SGLT2 expression, while anti-inflammatory cytokines FGF-21, IL-10 and TGF-β1 caused a decrease, compared with untreated control. This raises the possibility of a bidirectional relationship between SGLT2 and cytokine regulation.

This study has limitations, importantly the low sample size of the dapagliflozin-treated mouse group in proteomic analysis. This is a result of high volumes of plasma required for cytokine measurement and MS. Moreover, reduced function of SGLT2, as a result of altered gene expression in HK2 cells, was not confirmed. Future studies should include further interrogation of SGLT2 expression and functional regulation through glucose uptake detection in vitro, following treatment with different agents.

In summary, we found that genetic ablation of SGLT2 exerted more profound effects than pharmacological inhibition. Our data overall confirm previous studies reporting the effect of SGLT2 inhibition on cytokine secretion [43–45], which may be attributed to a number of mechanisms such as macrophage polarisation [9], T cell suppression [8] or resolution of low-grade inflammation through glycosuria [24]. However, SGLT2-null mice demonstrated a significantly improved metabolic phenotype, compared with SGLT2-inhibitor-treated mice. This effect was likely driven by a significant reduction in levels of proinflammatory cytokines, as well as an increase in anti-inflammatory cytokines, which subsequently led to physiological levels of corticosterone secretion.

As glycosuria, GLP-1 and body weight levels were similar between the two interventions, this observation opens questions on the physiological role of SGLT2 in immune response, the modulatory role of hyperglycaemia, and the temporal relationship between its metabolic effects and the anti-inflammatory effects reported in this study. Answering these questions could unravel a new mechanism of action for SGLT2 inhibitors and potentially a new avenue for the development of immunometabolic treatments.

## Supplementary Information

Below is the link to the electronic supplementary material.

ESM Table 1 (PDF 136 KB)

## Data Availability

The datasets generated during and/or analysed during the current study are available from the corresponding authors upon reasonable request.

## Abbreviations

11βHSD1: 11β-Hydroxysteroid dehydrogenase type 1
CAN: Acetonitrile
CBS: Central Biological Services
DEP: Differentially expressed protein
DIA: Data-independent acquisition
FA: Formic acid
FGF-21: Fibroblast growth factor 21
GLP-1: Glucagon-like peptide 1
GO: Gene ontology
HK2: Human kidney-2
HFD: High-fat diet
IL-6Rα: IL-6 receptor subunit α
NP: Nanoparticle
PCA: Principal component analysis
qPCR: Quantitative PCR
SGLT2: Sodium–glucose cotransporter 2
UGCR: Urinary glucose/creatinine ratio
WT: Wild-type
