# Supplementary material for: Effects of SGLT2 ablation or inhibition on corticosterone secretion in high-fat-fed mice: exploring a nexus with cytokine levels
Source: Diabetologia. 2025 Jun 20;68(9):2042–56. doi: 10.1007/s00125-025-06467-7 (PMC12361288; doi:10.1007/s00125-025-06467-7)
Supplement: Supplementary file 1 — ESM Table 1 (PDF 136 KB) [file 125_2025_6467_MOESM1_ESM.pdf]

Table 1: Primers used for the quantitative detection of transcripts normalized to β-actin

**Analysed transcript**	**Sequence of forward primer**	**Sequence of reverse primer**
Hsd11b1	GTTCGAAATCTTGAGGTTCTCTC	GGTGCCAGCAATGTAGTGTG
β-Actin	CACTGTCGAGTCGCGTCC	TCATCCATGGCGAACTGGTG
Slc5a2	GGCAGGCTCTGAACTTGGG	CCACAAGCCAACACCAATGACC
Il6	GCCTTCTTGGGACTGATGCT	TGTGACTCCAGCTTATCTCTTGG
Leptin (ob)	CATTTCACACACGCAGTCG	GACAGCCCAAAGGCTCTACC
Fgf21	GGGGGTCTACCAAGCATACC	CTTTGAGCTCCAGGAGACTTTCT
Il1β	TGCCACCTTTTGACAGTGATG	GTCTTGGCCGAGGACTAAGG
Il10	CCAGGTGAAGACTTTCTTTCAAAC	CTTGTAGACACCTTGGTCTTGGA
Tgfβ1	GCCACCTTTTGACAGTGATGAG	TTCTTGTGACCCTGAGCGAC
SLC5A2	GGACTCACAATCGTCTCGGG	GGCCTGGGGCTCATTCATC
β-ACTIN	GCACAGAGCCTCGCCTTT	CACAGGACTCCATGCCCAG
CYP11A1	CACGCTCAGTCCTGGTCAAA	CAGGCTGCCGACTTCTTCAA
HSD3B2	GCAGTAAGGACTTGGACTCCTC	CAACAGTAGCTGGGTACCTTTCA
